# Risk for Severe COVID-19 Outcomes among Persons with Intellectual Disabilities, the Netherlands

**DOI:** 10.3201/eid2901.221346

**Published:** 2023-01

**Authors:** Monique C.J. Koks-Leensen, Bianca W.M. Schalk, Esther J. Bakker-van Gijssel, Aura Timen, Masha E. Nägele, Milou van den Bemd, Geraline L. Leusink, Maarten Cuypers, Jenneken Naaldenberg

**Affiliations:** Radboud Institute for Health Sciences, Nijmegen, the Netherlands (M.C.J. Koks-Leensen, B.W.M. Schalk, E.J. Bakker–van Gijssel, A. Timen, M.E. Nägele, M. van den Bemd, G.L. Leusink, M. Cuypers, J. Naaldenberg);; Siza, Arnhem, the Netherlands (E.J. Bakker-van Gijssel);; Maastricht University Medical Center, Maastricht, the Netherlands (G.L. Leusink)

**Keywords:** COVID-19, respiratory infections, severe acute respiratory syndrome coronavirus 2, SARS-CoV-2, SARS, coronavirus disease, zoonoses, viruses, coronavirus, intellectual disability, long-term care, data register, case fatality, the Netherlands

## Abstract

The COVID-19 pandemic has disproportionately affected persons in long-term care, who often experience health disparities. To delineate the COVID-19 disease burden among persons with intellectual disabilities, we prospectively collected data from 36 care facilities for 3 pandemic waves during March 2020–May 2021. We included outcomes for 2,586 clients with PCR-confirmed SARS-CoV-2 infection, among whom 161 had severe illness and 99 died. During the first 2 pandemic waves, infection among persons with intellectual disabilities reflected patterns observed in the general population, but case-fatality rates for persons with intellectual disabilities were 3.5 times higher and were elevated among those >40 years of age. Severe outcomes were associated with older age, having Down syndrome, and having >1 concurrent condition. Our study highlights the disproportionate COVID-19 disease burden among persons with intellectual disabilities and the need for disability-inclusive research and policymaking to inform disease surveillance and public health policies for this population.

The global COVID-19 pandemic has had a disproportionate effect on persons in long-term care (*1*), particularly persons with intellectual disabilities (*2*). Persons with intellectual disabilities experience many limitations in adaptive behavior and intellectual functioning that occur before adulthood (*3*). Consequently, their ability to understand and adhere to restrictive measures is impaired. Social distancing is challenging for persons with intellectual disabilities living in group homes or during close contact when receiving care (*4*–*8*). In addition, genetic syndromes that cause intellectual disabilities, such as Down syndrome, might contribute to the susceptibility to and severity of COVID-19 (*7*–*12*). Persons with intellectual disabilities often have concurrent conditions, such as diabetes, cardiovascular problems, and being overweight (body mass index [BMI] >25 kg/m^2^) (*10*,*13*–*16*); they also are at increased risk for death from respiratory problems (*17*). Furthermore, COVID-19 pandemic risks can exacerbate health disparities among persons with intellectual disabilities (*2*,*18*).

Previous studies have shown substantially higher COVID-19 case rates, more hospital admissions, and higher case-fatality rates (CFRs) for persons with intellectual disabilities than for the general population, but those studies included relatively small sample sizes or were conducted during distinct periods of the pandemic (*6*,*9*–*11*,*19*–*22*). Besides the identified risk factors, intellectual disability also appeared to be an independent risk factor for severe COVID-19 outcomes, although the extent to which disability severity contributes is still unclear (*9*,*12*,*19*,*20*). Similarly, whereas pathogenicity of post–COVID-19 conditions is still emerging, specific characteristics among persons with intellectual disabilities and persistent post–COVID-19 symptoms are potentially unrecognized and unclear (*23*).

Because population surveillance for COVID-19 does not include information about disabilities, complete and integrated information about this vulnerable subgroup is lacking and potentially contributing to growing health disparities. To delineate specific factors driving excess risks for persons with intellectual disabilities infected with SARS-CoV-2, more information on the dynamic course of the outbreak, risk factors such as concurrent conditions, and population health in the local context is urgently needed. 

We used data from a prospective nationwide registry on persons with intellectual disabilities and COVID-19 in long-term care in the Netherlands to provide comprehensive insight into the COVID-19 disease burden among this population. We aimed to examine characteristics of persons with intellectual disabilities and COVID-19, stratified by outcome severity; describe the course of SARS-CoV-2 infection and death in persons with intellectual disabilities across the initial 3 COVID-19 pandemic waves; and explore associations between severe outcomes and patient characteristics.

## Methods

### Design and Setting

We conducted an observational registry-based prospective study by collecting data on residents and outpatients with suspected COVID-19 from long-term care organizations in the Netherlands. The registry was a joint initiative of specialized intellectual disabilities physicians, researchers, and the Ministry of Health, Welfare, and Sport of the Netherlands to establish an adequate basis for policy and practice decision making regarding COVID-19 among persons with intellectual disabilities. We invited all 170 member organizations of Vereniging Gehandicaptenzorg Nederland, the association for disability care in the Netherlands, to participate; we also opened participation to nonmember organizations. In all, 36 organizations across the Netherlands participated, serving ≈60% of the estimated 115,000 clients with intellectual disabilities in long-term care (*24*). We considered the organizations geographically representative, which was necessary to adequately compare with general population data considering differences in regional spread of SARS-CoV-2. The registry was open from March 24, 2020–September 1, 2021. We collected and included data from cases that occurred during March 24, 2020–June 1, 2021, and facilities could enter follow-up data until September 1, 2021. The Medical Research Ethics Committee of Radboud University Medical Center approved the study without need for informed consent because this was a minimal risk study with de-identified data (reference no. 2020-6509).

### Data Collection and Outcome Measures

Each participating location was granted access to an online registration system (Castor, https://www.castoredc.com). For each patient with intellectual disabilities suspected of COVID-19, participating organizations completed a questionnaire concerning demographic characteristics (age, sex, residential status), medical history (etiology and severity level of disability, concurrent conditions, and medications), and test status. For patients with COVID-19 confirmed by a positive PCR test, we obtained additional information regarding the need for oxygen therapy, hospital admission, and whether the patient died. Questions had a categorical or dichotomous answering scale, with an option to add free text when other was selected in a category (Appendix).

This study only included patients with a COVID-19 diagnosis, which we defined as a positive SARS-CoV-2 PCR test result during the study period. Our primary outcomes were serious COVID-19 illness or death within 4 weeks of COVID-19 diagnosis. We defined serious illness as a need for oxygen therapy, considered or actual hospital admission for COVID-19, or both. We assumed mild COVID-19 disease for all patients who did not experience severe illness or death. We registered reinfections by updating entries for patients after a record was opened, providing additional test data, and adding information for the COVID-19 case. However, we only included the first confirmed infection for each patient in this study. We retrieved comparator data for the general population of the Netherlands during the study period from publicly available data of the Rijksinstituut voor Volksgezondheid en Milieu (RIVM), the National Institute for Public Health and the Environment, which is responsible for population monitoring of COVID-19 in the Netherlands (*25*).

### Wave Definition

In response to the different COVID-19 waves, testing and preventive regulations changed over the course of the pandemic. For comparability, we followed the same start and end dates per wave, which RIVM identified on the basis of SARS-CoV-2 infections in the general population. Wave I ran from epidemiologic week 11, 2020 through week 25, 2020; wave II ran from week 26, 2020, through week 4, 2021; and wave III ran from weeks 5 through 21, 2021 (*26*). We assigned patients in our study to a pandemic wave on the basis of reported date of positive PCR test. For cases missing PCR testing dates, we used the date of reported illness onset instead.

### Statistical Methods

For descriptive characteristics, we used frequency and percentage or median and interquartile range **(**IQR) for the entire study population and stratified characteristics by outcomes as mild illness, severe illness, or death. We excluded patients with missing information on both test date and first date of illness from comparison between waves because we could not assign them to a specific wave. We separately calculated the CFR per wave by sex and age group (0–17 years, 18–39 years, 40–69 years, and >70 years) by using the number of reported deaths as numerator and the total number of confirmed infections as denominator. We also calculated rates for serious illness and mild illness by dividing the number of serious or mild cases by the total number confirmed infections in the study population per wave. We used the same calculations to compare illness and death rates for general population data for the same strata.

To examine associations between demographic characteristics and concurrent conditions (dependent factors) and severe COVID-19 illness and death as outcomes, we conducted logistic regression modeling. In a first step, we assessed effects of sex, age, disability level, Down syndrome, and concurrent conditions by using a univariable model for each separate outcome measure to assess relevant variables for multivariate analysis and considered p<0.10 statistically significant. We combined all variables with a significant univariate association in the multivariable model. We conducted stepwise backward logistic regression with a significance level for removing variables of 0.10 (p value out) from the full model and for re-entering variables as 0.05 (p value in). We calculated the odds ratio (OR) and 95% CI for potential risk factors for severe outcomes. We used receiver operating characteristic area under the curve (AUC) to evaluate predictive performance of the multivariable models. AUC uses a combination of sensitivity and specificity of model predictions and actual cases of severe illness or death, to assess predictive performance. An AUC of 0.50 indicates no predictive ability, and higher values correspond to better performance. We assessed adequate model fit by using Hosmer-Lemeshow goodness-of-fit tests on both multivariable models and accepted cases in which p>0.05. We used 2-sided statistical tests for all calculations and considered p<0.05 statistically significant. We tested for collinearity among all independent variables by using the variance inflation factor (VIF) and retained covariates for each final analysis that had a VIF <5. We conducted all statistical analyses in SPSS Statistics 25.0 (IBM, https://www.ibm.com).

## Results

Data for 9,163 persons with intellectual disabilities suspected of COVID-19 were entered into the registry, of which 2,586 (28.2%) had a PCR-confirmed SARS-CoV-2 infection. For 161 (6.2%) of these patients, severe illness was reported, and 99 (3.8%) patients died after their SARS-CoV-2 infection.

### Characteristics of Persons with Intellectual Disabilities and COVID-19

We assessed demographic and health condition characteristics of 2,586 persons with intellectual disabilities and COVID-19, including their illness outcomes (Table 1). The median age was 51 (IQR 34–62) years, most (58.5%, n = 1,476) patients were men, and most (79.9%, n = 2,067) lived in group homes. Disability severity had equal representation, and 176 (6.8%) patients had Down syndrome. Among all included patients, 1,101 (42.6%) had concurrent conditions. The most prevalent conditions were being overweight (26.2%, n = 678), epilepsy (10.4%, n = 268), hypertension (7.5%, n = 195), diabetes (5.8%, n = 151), and chronic heart disease (4.6%, n = 120).

**Table 1:** Characteristics and outcomes for 2,586 persons included in a study of risk for severe COVID-19 outcomes among persons with intellectual disabilities, the Netherlands*

Characteristics	Mild illness, n = 2,326	Severe illness, n = 161	Died, n = 99†	Total, n = 2,586
Sex, no. (%), n = 2,525				
M	1,315 (58.0)	103 (64.0)	58 (59.2)	1,476 (58.5)
F	951 (42.0)	58 (36.0)	40 (40.8)	1,049 (41.5)
Median age, y (IQR), n = 2,519	49 (32.0–61.0)	61 (52.0–67.5)	68 (61.0–76.0)	51 (34–62)
Age groups, no. (%), n = 2,519				
0–17 y	81 (3.6)	0	1 (1.0)	82 (3.3)
18–39 y	721 (31.9)	16 (9.9)	2 (2.0)	739 (29.3)
40–49 y	330 (14.6)	16 (9.9)	4 (4.0)	350 (13.9)
50–59 y	491 (21.7)	44 (27.3)	16 (16.2)	551 (21.9)
60–69 y	399 (17.7)	57 (35.4)	32 (32.3)	488 (19.4)
>70 y	237 (10.5)	28 (17.4)	44 (44.4)	309 (12.3)
Long term care type, no. (%)				
Group home	1,853 (79.7)	132 (82.0)	82 (82.8)	2,067 (79.9)
Independent living	349 (15.0)	28 (17.4)	16 (16.2)	393 (15.2)
Other or unknown	124 (5.3)	1 (0.6)	1 (1.0)	126 (4.9)
Disability level, no. (%), n = 2,468				
Borderline to mild	632 (28.5)	49 (31.2)	18 (18.9)	699 (28.3)
Moderate	798 (36.0)	47 (29.9)	42 (44.2)	887 (35.9)
Severe to profound	786 (35.5)	61 (38.9)	35 (36.8)	882 (35.7)
Disability etiology, no. (%)				
Down syndrome	141 (6.1)	20 (12.4)	15 (15.2)	176 (6.8)
No. concurrent conditions (%)				
None reported	1,383 (59.5)	62 (38.5)	40 (40.4)	1,485 (57.4)
1 reported	650 (27.9)	54 (33.5)	31 (31.3)	735 (28.4)
>1 reported	293 (12.6)	45 (28.0)	28 (28.3)	366 (14.2)
Concurrent conditions, no. (%)				
Diabetes	117 (5.0)	19 (11.8)	15 (15.2)	151 (5.8)
Hypertension	159 (6.8)	20 (12.4)	16 (16.2)	195 (7.5)
Heart disease	95 (4.1)	11 (6.8)	14 (14.1)	120 (4.6)
Lung disease; asthma, COPD, or both	57 (2.5)	13 (8.1)	9 (9.1)	79 (3.1)
Epilepsy	229 (9.8)	25 (15.5)	14 (14.1)	268 (10.4)
Overweight, body mass index >25 kg/m^2^	587 (25.2)	67 (41.6)	24 (24.2)	678 (26.2)

*All persons included in the study had intellectual disabilities and tested positive for SARS-CoV-2 by PCR. Category totals do not always add up to column totals because of missing responses; percentages are based on variable totals per category. COPD, chronic obstructive pulmonary disease; IQR, interquartile range. †Case-fatality ratio 3.8%.

Patients with severe illness and those who died were older than others in the entire sample. The median age of persons with severe illness was 61 (IQR 52–67.5) years, and for those who died, median age was 68 (IQR 61–76) years. Those subgroups also included higher percentages of patients with Down syndrome, 12.4% (n = 20) of patients with severe illness and 15.2% (n = 15) of patients who died. In addition, approximately two thirds of patients who had severe illness (61.5%, n = 99) or who died (59.6%, n = 59) had concurrent conditions, compared with only 40.5% (n = 943) of patients who had mild illness (Table 1).

### Infections and Outcomes Per Wave

**Figure 1:**
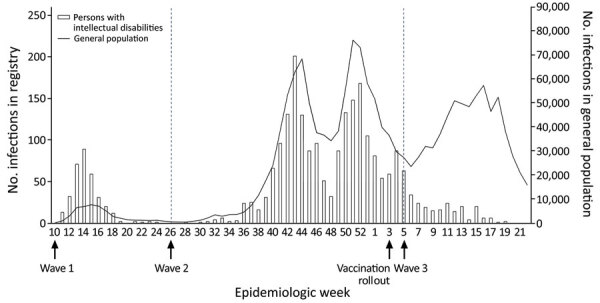
Weekly number of COVID-19 infections among persons with intellectual disabilities and the general population, the Netherlands, March 2020–May 2021. Graph shows epidemiologic weeks during 3 pandemic waves in the Netherlands: wave I, March–June 2020; wave II, July 2020–January 2021; and wave III, February–May 2021. The registry included 2,586 persons with intellectual disabilities in long-term care. Scales for the y-axes differ substantially to underscore patterns.

The first wave of COVID-19 included 335 patients with intellectual disabilities, the second wave 1,927 patients, and the third wave 268 patients (Table 2). The pattern in weekly infections among persons with intellectual disabilities followed similar patterns as those for the general population for the first 2 waves and declined with the start of the vaccination campaign during the third wave (Figure 1). During the first wave, 17.1% (n = 57) of patients were >70 years of age, which is more than in subsequent waves: 11.6% (n = 221) in the second wave and 11.7% (n = 31) in the third wave. COVID-19 among younger persons, those 0–39 years of age, increased from 1.8% (n = 6) in the first wave to 6.4% (n = 17) in the third wave for those aged 0–17 years and from 20.4% (n = 68) in the first wave to 28.9% (n = 77) in the third wave for those 18–39 years of age (Table 2).

**Table 2:** Outcomes per COVID-19 wave among persons included in a study of risk for severe COVID-19 outcomes among persons with intellectual disabilities, the Netherlands*

Outcomes	Wave I, March–June 2020	Wave II, July 2020–January 2021	Wave III, February–May 2021
Total COVID-19 infections	335	1,927	268
Sex, no. (%)			
M	178 (53.1)	1,138 (59.5)	153 (57.1)
F	157 (46.9)	776 (40.5)	115 (42.9)
Infections per age group, no. (%)			
0–17 y	6 (1.8)	58 (3.0)	17 (6.4)
18–39 y	68 (20.4)	593 (31.0)	77 (28.9)
40–69 y	202 (60.7)	1,039 (54.4)	141 (53.0)
>70 y	57 (17.1)	221 (11.6)	31 (11.7)
Mild illness, no. (%)†	240 (71.6)	1,785 (92.6)	247 (92.2)
Sex, no. (%)			
M	126 (52.5)	1,039 (58.6)	144 (58.3)
F	114 (47.5)	733 (41.4)	103 (41.7)
Infections per age group, no. (%)			
0–17 y	6 (2.5)	57 (3.2)	17 (6.9)
18–39 y	64 (26.9)	579 (32.7)	77 (31.4)
40–69 y	139 (58.4)	948 (53.6)	128 (52.2)
>70 y	29 (12.2)	185 (10.5)	23 (9.4)
Severe illness, no. (%)	46 (13.7)	99 (5.1)	14 (5.2)
Sex, no. (%)			
M	27 (58.7)	68 (68.7)	7 (50.0)
F	19 (41.3)	31 (31.3)	7 (50.0)
Infections per age group, no. (%)			
0–17 y	0	0	0
18–39 y	3 (6.5)	13 (13.1)	0
40–69 y	34 (73.9)	71 (71.7)	10 (71.4)
>70 y	9 (19.6)	15 (15.2)	4 (28.6)
No. deaths (case-fatality ratio)‡	49 (14.6)	43 (2.2)	7 (2.6)
Sex, no. (%)			
M	25 (52.1)	31 (72.1)	2 (28.6)
F	23 (47.9)	12 (27.9)	5 (71.4)
Deaths per age group, no. (%)			
0–17 y	0	1 (2.3)	0
18–39 y	1 (2.0)	1 (2.3)	0
40–69 y	29 (59.2)	20 (46.5)	3 (42.9)
>70 y	19 (38.8)	21 (48.8)	4 (57.1)

*Because of missing data for some responses, values might not add up to 100%. For missing SARS-CoV-2 testing date, we used reported date of illness onset instead. In total, 56 patients had no date information and could not be assigned to a specific wave, of which 54 reported mild illness and 2 reported severe illness. †Missing data for sex, n = 13, and age, n = 20. ‡Missing data for sex, n = 1.

Severe illness was highest during the first wave (13.7%, n = 46) and was comparable during the second (5.1%, n = 99) and third (5.2%, n = 14) waves. In all 3 waves, rates of severe illness were highest (71.4%–73.9%) among patients 40–69 years of age (Table 2).

**Figure 2:**
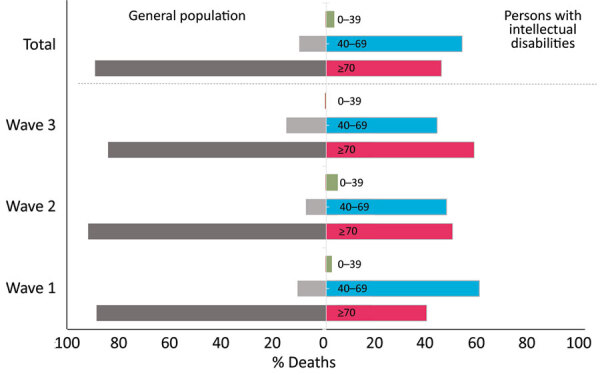
Distribution of COVID-19 deaths across age groups among persons with intellectual disabilities and the general population during 3 pandemic waves, the Netherlands. Wave I was March–June 2020; wave II, July 2020–January 2021; and wave III, February–May 2021. Information on 2,586 persons with intellectual disabilities was collected from long-term care organizations that care for this population.

CFR decreased from 14.6% during the first wave to 2.2% during the second wave and 2.6% in the third wave. Across all 3 waves, the CFR was 3.8% among our study cohort, whereas overall CFR was only 1.1% in the general population of the Netherlands (*25*). Among persons with intellectual disabilities, a substantial number of deaths occurred among persons between 40–69 years of age, whereas death in the general population was concentrated among persons >70 years of age (Figure 2).

### Factors Associated with Severe COVID-19 Illness and Death

In multivariable analysis, we found that severe illness was more likely for patients with Down syndrome (OR 2.6, 95% CI 1.5–4.3) and for patients with several concurrent conditions, including lung diseases (OR 3.5, 95% CI 1.8–6.7), diabetes mellitus (OR 1.8, 95% CI 1.0–3.0), epilepsy (OR 1.8, 95% CI 1.1–2.8), or who were overweight (OR 1.8, 95% CI 1.3–2.5) (Table 3). Age was also substantially associated with severe COVID-19 illness and risks increased with increasing age (OR 1.04, 95% CI 1.03–1.05).

**Table 3:** Univariable and multivariable logistic regression for severe illness by characteristics among 161 persons with intellectual disabilities and COVID-19, the Netherlands*

Characteristics	Univariable			Multivariable†	
	Odds ratio (95% CI)	p value		Odds ratio (95% CI)	p value
Sex					
M	1.3 (0.9–1.8)	0.14		ND	NA
F	Referent				
Age	1.04 (1.0–1.1)	<0.001		1.04 (1.03–1.05)	<0.001
Disability level					
Borderline to mild	Referent				
Moderate	1.0 (0.7–1.5)	0.99		ND	NA
Severe to profound	1.3 (0.9–2.0)	0.19		ND	NA
Etiology					
Down syndrome	2.2 (1.3–3.6)	0.002		2.6 (1.5–4.3)	<0.001
Concurrent conditions					
Diabetes	2.5 (1.5–4.2)	<0.001		1.8 (1.0–3.0)	0.04
Hypertension	1.9 (1.2–3.2)	0.009		ND	NA
Heart disease	1.7 (0.9–3.3)	0.10		ND	NA
Lung disease	3.5 (1.9–6.5)	<0.001		3.5 (1.8–6.7)	<0.001
Epilepsy	1.7 (1.1–2.6)	0.02		1.8 (1.1–2.8)	0.02
Overweight, BMI >25 kg/m^2^	2.1 (1.5–2.9)	<0.001		1.8 (1.3–2.5)	<0.001

*Because of nonresponses for some patient data among 2,586 persons included in the study, these data reflect missing values for sex, n = 60; age n = 67; and disability level, n = 114. BMI, body mass index; NA, not applicable; ND, not done. †Variables with p<0.1 in univariable analyses were included in multivariate logistic regression analysis. Because we used stepwise backward selection, we removed nonsignificant variables from the multivariable model and we could not provide estimates. The area under the curve was 0.731 (95% CI 0.691–0.770; p<0.001). We used Hosmer-Lemeshow goodness-of-fit test to assess the model fit for logistic regression and considered p>0.05 nonsignificant. Variance inflation factor (VIF) diagnostics indicated no evidence of collinearity (all VIF<1.2) among variables in final model.

We performed similar logistic regressions for COVID-19–related deaths (Table 4). Increased risk of COVID-19 death was associated with increasing age (OR 1.1, 95% CI 1.1–1.1), and having Down syndrome (OR 5.6, 95% CI 2.9–10.6), lung disease (OR 4.6, 95% CI 2.0–10.7), or heart disease (OR 2.3, 95% CI 1.2–4.5).

**Table 4:** Univariable and multivariable logistic regression by characteristics among 99 persons with intellectual disabilities who died of COVID-19, the Netherlands*

Characteristics	Univariable			Multivariable†	
	Odds ratio (95% CI)	p value		Odds ratio (95% CI)	p value
Sex					
M	1.0 (0.7–1.6)	0.82		ND	
F	Referent				
Age	1.1 (1.1–1.1)	<0.001		1.09 (1.07–1.12)	<0.001
Disability level‡					
Borderline to mild	Referent				
Moderate	0.5 (0.3–0.9)	0.03		ND	NA
Severe to profound	0.6 (0.4–1.1)	0.13		ND	NA
Disability etiology‡					
Down syndrome	2.86 (1.6–4.9)	0.001		5.6 (2.9–10.6)	<0.001
Concurrent conditions					
Diabetes	3.4 (1.9–6.0)	<0.001		ND	NA
Hypertension	2.6 (1.5–4.6)	0.001		ND	NA
Heart disease	3.9 (2.1–7.1)	<0.001		2.3 (1.2–4.5)	0.01
Lung disease	4.0 (1.9–8.3)	<0.001		4.6 (2.0–10.7)	<0.001
Epilepsy	1.5 (0.8–2.7)	0.17		ND	NA
Overweight, BMI >25 kg/m^2^	0.9 (0.6–1.5)	0.82		ND	NA

*Because of nonresponses for some patient data among 2,586 persons included in the study, these data reflect missing values for sex, n = 60; age n = 67; and disability level, n = 114. BMI, body mass index; NA, not applicable; ND, not done. †Variables with p<0.1 in univariable analyses were included in multivariate logistic regression analysis. Because we used stepwise backward selection, we removed nonsignificant variables from the multivariable model and we could not provide estimates. The area under the curve was 0.844 (95% CI 0.808–0.880; p<0.001). We used Hosmer-Lemeshow goodness-of-fit test to assess the model fit for logistic regression and considered p>0.05 nonsignificant. Variance inflation factor (VIF) diagnostics indicated no evidence of collinearity (all VIF<1.2) among variables in final model. ‡Both the variable disability level and the variable etiology concern the level of intellectual disability. To avoid interdependency, we only included etiology in the multivariable model, because this variable shows a stronger univariable relationship and had no missing values.

## Discussion

We report outcomes of a nationwide prospective COVID-19 registry of persons with intellectual disabilities in long-term care in the Netherlands during March 2020–May 2021. This registry provided a large dataset of COVID-19–positive patients with intellectual disabilities collected during 15 consecutive months of the pandemic. In addition to national surveillance data about the general population, this prospective registry generated detailed insights into COVID-19 disease and risk factors among the subpopulation of persons with intellectual disabilities.

COVID-19 among persons with intellectual disabilities followed similar epidemiologic wave patterns as those for the general population for the first 2 pandemic waves in the Netherlands, indicating the difficulty of protecting vulnerable subpopulations from generic contamination routes. The observed third wave of COVID-19 in persons with intellectual disabilities was less pronounced, which could be an indication of COVID-19 vaccine effectiveness in this subpopulation. Large-scale vaccination roll-out in the Netherlands started at the onset of the third wave and prioritized persons with intellectual disabilities along with other risk groups. Despite the rather similar epidemiologic pattern of COVID-19 in the general population and in persons with intellectual disabilities, pronounced differences were seen in the clinical course of the disease and its outcomes.

In our study population, we found the COVID-19 CFR was >3 times higher for persons with intellectual disabilities than for the general population of the Netherlands at a given time (*25*). In contrast to the general population, most deaths among persons with intellectual disabilities occurred at relatively young ages (40–69 vs. >70 years of age). Those findings are consistent with reports from the United Kingdom (*12*,*19*,*21*), Canada (*11*), and the United States (*4*,*9*,*10*,*20*), which implies that age-related thresholds applied to the general population in protective policies require adjustment when applied to the intellectual disability population.

In line with previous findings, patients with severe COVID-19 outcomes in our registry were older, more frequently had Down syndrome, and had a larger percentage reporting >1 concurrent condition compared with patients facing mild illness. In addition to studies reporting effects of intellectual disability level as a risk factor (*12*,*16*), we did not find notable effects associated with disability severity. Our results indicate that several conditions were associated with risk for severe illness and death; chronic heart disease and lung diseases (asthma, COPD, or both) were significantly associated with COVID-19–related deaths (p<0.001), and having diabetes, epilepsy, or lung disease or being overweight increased risk for severe COVID-19 illness. One previous study also identified heart disease as a risk factor for COVID-19–related death among persons with intellectual disabilities (*10*). Other concurrent conditions we included in our analyses did not show statistically significant associations with COVID-19 death in our within-group analyses, although conditions such as diabetes, epilepsy, and being overweight are generally reported to be risk factors for COVID-19–related death, hospitalization, or both, and were relatively common in our entire sample of SARS-CoV-2–positive patients (*19*,*20*,*27*). However, clinicians should recognize the associations between underlying health conditions and severe COVID-19 outcomes reported here to ensure that persons with intellectual disabilities and concurrent conditions receive appropriate medical care.

Future efforts to protect persons with intellectual disabilities in long-term care settings from adverse outcomes during this pandemic and future pandemics need to balance between protection and effects of implemented measures and restrictions, accounting for vulnerabilities and increased disease burden among this population. Accurate data to support decision making are then required. An example of policy implications of our national registry is that it provided supportive evidence to prioritize vaccination for persons with intellectual disabilities in the Netherlands. Large-scale vaccination rollout started earlier for persons with intellectual disabilities than for the general population, resulting in less severe SARS-CoV-2 infections and consecutive gradual relaxations of socially restrictive measures in this population.

A strength of our study is collection of specific data from a representative sample of long-term care providers in the Netherlands that could not be retrieved from other sources. Of note, our registry was affected by changes in testing protocols. During the first wave, testing was available only under certain conditions for symptomatic patients, resulting in an overrepresentation of severe cases and a higher CFR among both groups.

However, one consequence of our registration method was that it did not provide information about the total population of persons with intellectual disabilities to which reported COVID-19 cases related. Therefore, we could not estimate the incidence of infections and death for the intellectual disability population at large, and we only had complete information to calculate CFRs within our sample. Furthermore, we observed no effect from residential status, probably because our data collection method focused on intellectual disability care facilities providing long-term care, which predominantly comprises residential care. The prevalence of some other risk factors was too low to include in analyses and obtain a complete profile of all potentially relevant risk factors. Although we had a large registry and total study population of persons with intellectual disabilities and COVID-19, the numbers of observations for some of the variables in our multivariable logistic models were low. Because OR and 95% CI provide a clear direction of the observed associations, we do not assume the small sample size substantially influenced our results. 

To gain more accurate insights into risks associated with concurrent conditions, research incorporating control groups of persons without intellectual disabilities and without COVID-19 is needed to enable comparisons between groups. Finally, potential selection bias cannot be excluded because of a greater perceived relevance of reporting severe cases. Our study comprised the initial 3 pandemic waves and did not enable long-term follow-up to quantify the occurrence of post–COVID-19 syndromes. Long-term follow-up studies in persons with intellectual disabilities could provide further insights.

The findings from our prospective registry-based data provide critical information about risk factors and health disparities among persons with intellectual disabilities obscured in national surveillance data. In addition, the results contribute to the disability-inclusive response in research, policy, and practice that is currently called upon and will be needed in future pandemics. We collected specific information directly from care providers to demonstrate COVID-19 disease burden and factors affecting disease progression within the persons with intellectual disabilities group. Our data show persons with intellectual disabilities are a risk group that requires dedicated monitoring and evidence-based policies. Epidemiologic evidence of the COVID-19 disease burden among persons with intellectual disabilities is essential for addressing knowledge gaps and informing adequate policymaking. Our results highlight the specific need for attention to this group in policymaking to prevent growing inequities and provide quality care during pandemics. 

Appendix Data collection tool used in study of risk for severe COVID-19 outcomes among persons with intellectual disabilities, the Netherlands.
