# Supplementary material for: Risk for Severe COVID-19 Outcomes among Persons with Intellectual Disabilities, the Netherlands
Source: Emerg Infect Dis. 2023 Jan;29(1):118–26. doi: 10.3201/eid2901.221346 (PMC9796201; doi:10.3201/eid2901.221346)
Supplement: Appendix — Data collection tool used in study of risk for severe COVID-19 outcomes among persons with intellectual disabilities, the Netherlands. [file 22-1346-Techapp-s1.pdf]

Article DOI: https://doi.org/10.3201/eid2901.221346

Risk for Severe COVID-19 Outcomes among Persons with Intellectual Disabilities, the Netherlands

Appendix

Data Collection Tool for Persons with Intellectual Disabilities and COVID-19 in Long-Term Care

1. Sociodemographics

- Sex: male / female / other
- Age in years
- Level of intellectual disability: borderline / mild / moderate / severe / profound
- Living situation: independent / group home / other
- Number of people living in the house
- Location and setting of the house
- Name of the living facility

2. COVID-19 infection

- Date of testing
- Date of reporting sick
- Date of first COVID-19 related symptoms
- Symptoms – Fever (≥38°C): no / yes / unknown
- Symptoms – Cough: no / yes / unknown
- Symptoms – Sore throat: no / yes / unknown

- Symptoms – Shortness of breath: no / yes / unknown
- Symptoms – Nasal cold / sneezing: no / yes / unknown
- Symptoms – Fatigue: no / yes / unknown
- Symptoms – Cough up sputum: no / yes / unknown
- Symptoms – Headache: no / yes / unknown
- Symptoms – Gastrointestinal complaints: no / yes / unknown
- Symptoms – Other: no / yes / unknown. If yes, what other symptoms (open text)
- Possible source of contamination: fellow client / care provider / parent / other / unknown
- Extra oxygen requirement: no / yes / unknown
- Hospital admission deemed necessary: no / yes / unknown
    - o If yes: reason for not admitting to hospital
- Deceased; no / yes
    - o If yes: date of death

3. Medical History

- Etiology of the intellectual disability (if known)
- Height in cm
- Weight in kilograms
- Body Mass Index (automatically calculated)
- Smoking status: current smoker / former smoker / never smoker / unknown
- Chronic heart disease: no / yes / unknown
    - o If yes: congenital heart disease: no / yes / unknown
    - o If yes: what congenital heart disease (open text)
- Other chronic conditions: hypertension / diabetes mellitus / asthma / COPD / kidney disease leading to dialysis or kidney transplant / other / unknown

o If other: what chronic condition (open text)

4. Other

All other relevant and notable remarks or observations that might affect COVID-19 outcomes should be mentioned (open text)
